# Supplementary figures and images for: Protective function and durability of mouse lymph node-resident memory CD8+ T cells
Source: eLife. 2021 Jun 18;10:e68662. doi: 10.7554/eLife.68662 (PMC8213409; doi:10.7554/eLife.68662)

## Slide 1
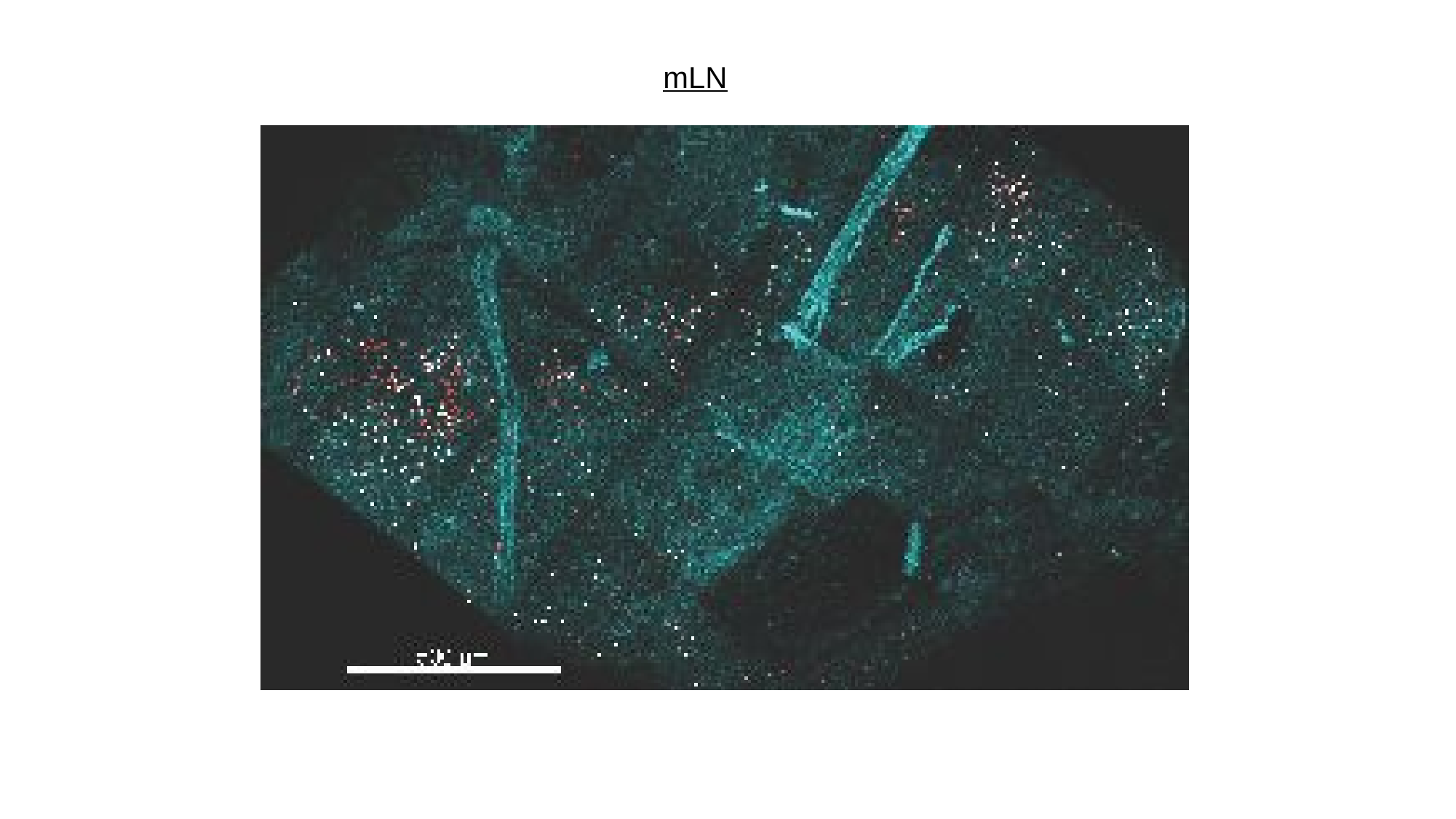

mLN

## Slide 2
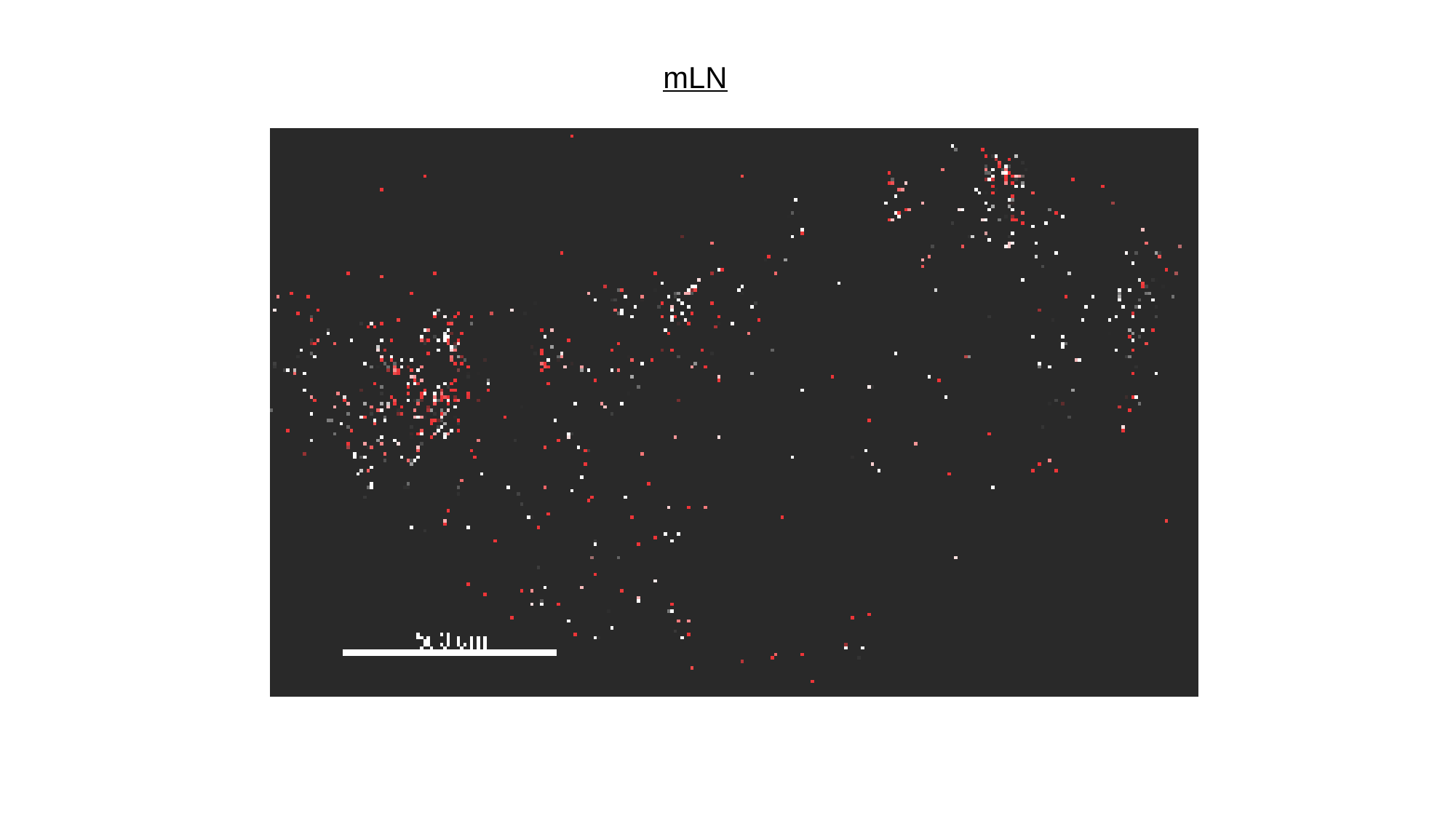

mLN

## Slide 3
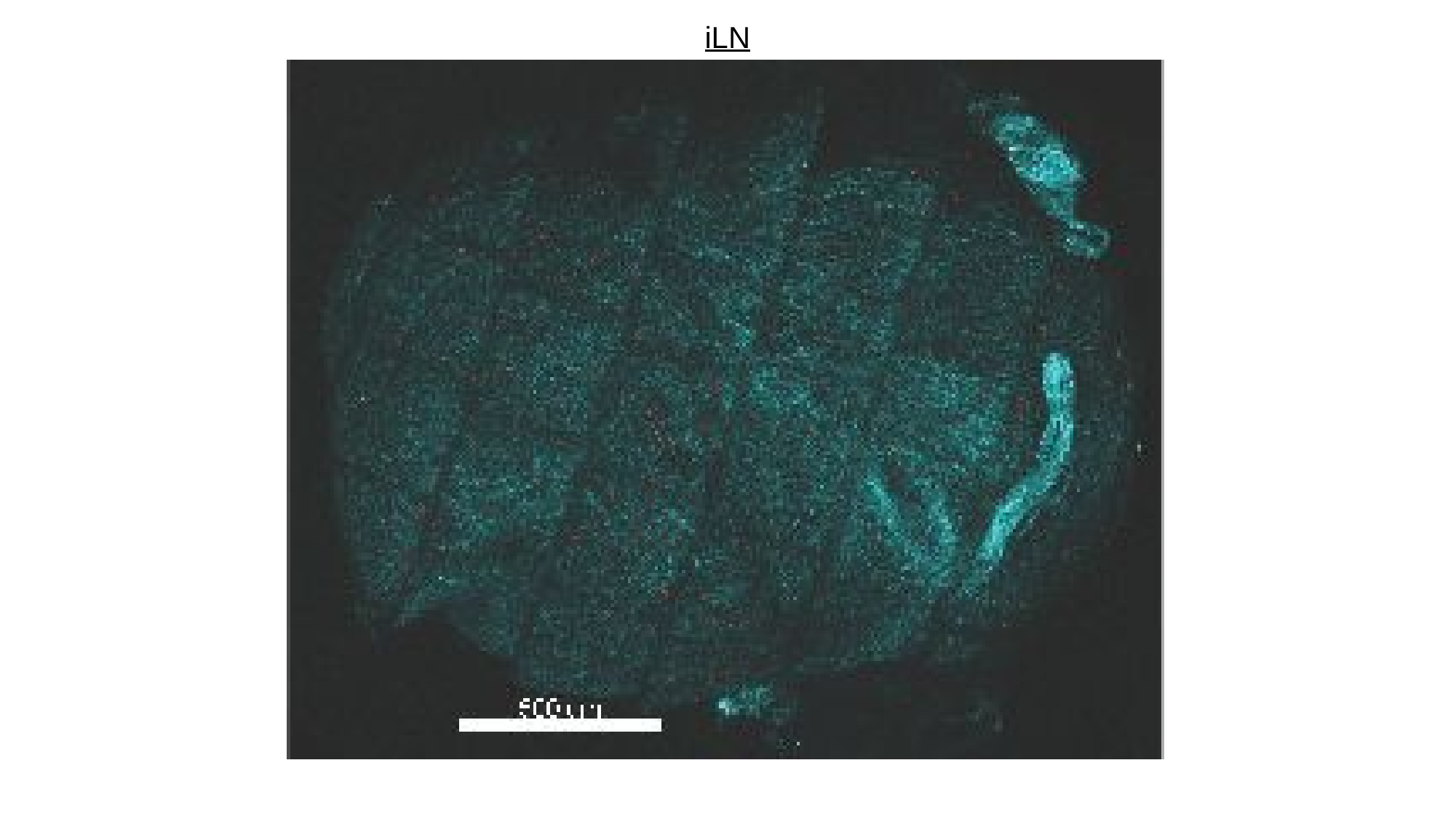

iLN

## Slide 4
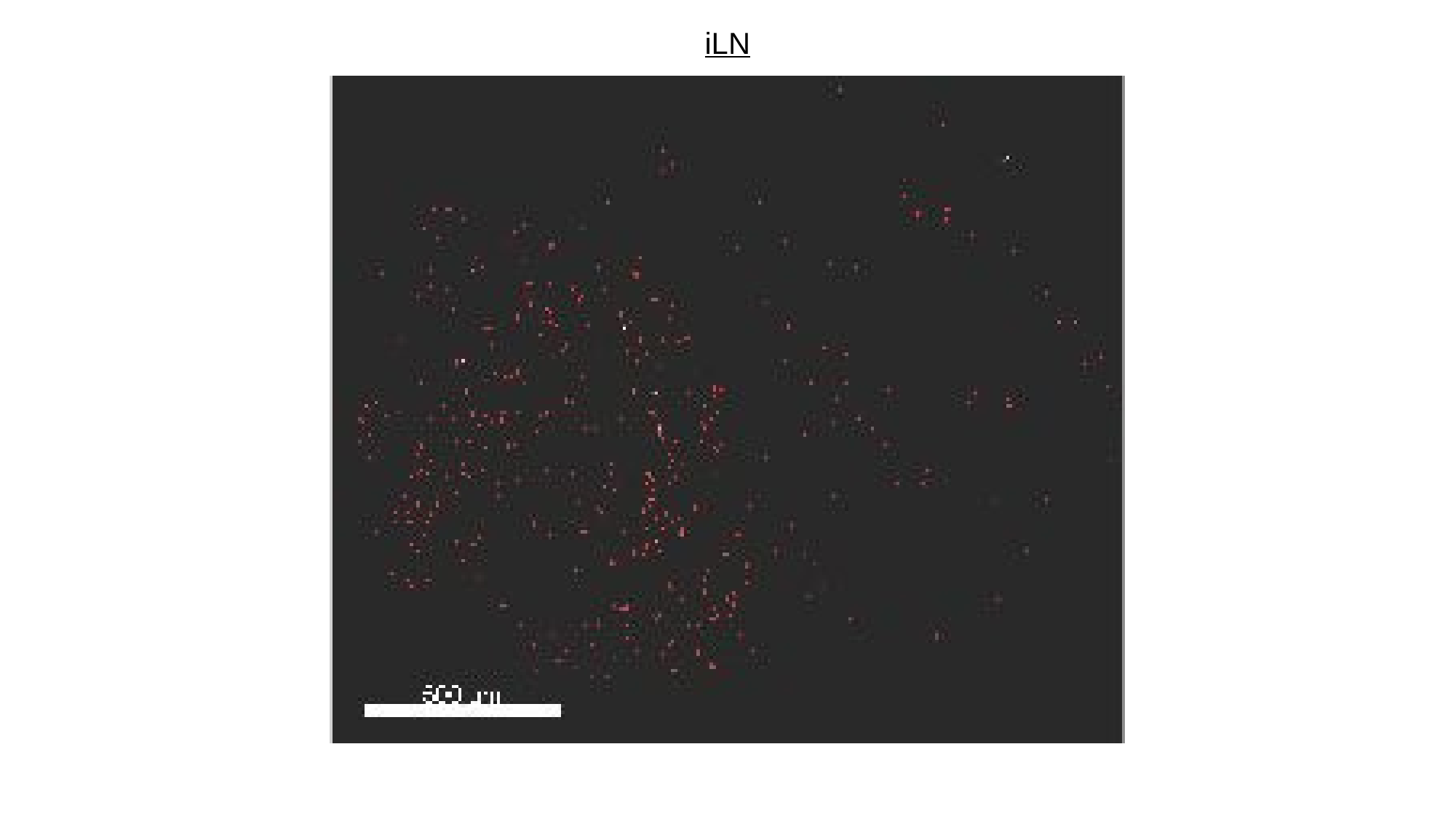

iLN

Supplement: Figure 5—source data 1. [file elife-68662-fig5-data1.pptx]
